# Unraveling Cytomegalovirus Drug Resistance in Transplant Patients by Targeting Deep Sequencing

**DOI:** 10.1002/jmv.70768

**Published:** 2025-12-29

**Authors:** Salvador Alemán, Juan Camacho, Vanessa Recio, Estrella Ruiz, Pilar Zamarrón, Jorge Anel, Montserrat Enjuto, David Tarragó

**Affiliations:** ^1^ Centro Nacional de Microbiología, Instituto de Salud Carlos III Madrid Spain; ^2^ Hospital Universitario Puerta de Hierro Madrid Spain; ^3^ Werfen Group, L′Hospitalet de Llobregat Barcelona Spain; ^4^ Institut Botànic de Barcelona, Consejo Superior de Investigaciones Científicas Barcelona Spain

**Keywords:** ARM, CMV viral load, NGS, Sanger sequencing, transplant patients

## Abstract

Drug‐resistant cytomegalovirus (CMV) poses a major clinical challenge in transplant recipients, leading to treatment failure and increased morbidity. This study applied a next‐generation sequencing (NGS) approach to identify antiviral resistance mutations (ARMs) in 71 samples from 68 CMV‐positive patients who had undergone hematopoietic stem cell transplantation (HSCT) or solid organ transplantation (SOT) between 2018 and 2024. A custom nested‐PCR protocol targeting six CMV genes (UL27, UL51, UL54, UL56, UL89, and UL97) was developed for enrichment prior to NGS. ARMs were detected in 23% of patients without clinical suspicion of resistance and in 62% of those with suspected resistance, most frequently affecting UL97. The most common UL97 mutations were A594V (24.4%), C603W (20.0%), and L595S (15.6%), while D301N (50%) predominated in UL54. Mutations associated with foscarnet and maribavir resistance were found in five and eight patients, respectively. NGS identified ARMs in 29 patients not detected by Sanger sequencing (*p* < 0.00001), while no additional ARMs were identified by Sanger alone. Importantly, these minority variants, revealed by NGS, are clinically relevant, as they may expand under antiviral pressure and contribute to virological failure. ARM presence was not significantly associated with viral load or mortality, though recurrent CMV reactivation showed a trend toward association (*p* = 0.0504). Survival was significantly lower in HSCT versus SOT recipients (*p* = 0.027). These findings support the routine clinical use of NGS for CMV resistance testing, particularly in complex cases and in the context of expanding antiviral options such as maribavir and letermovir.

## 1 Introduction

Transplant recipients face significant challenges in managing cytomegalovirus (CMV) infection due to immunosuppression and the emergence of antiviral resistance caused by prolonged antiviral exposure. Genotypic resistance assays are critical for guiding therapy in hematopoietic stem cell transplant (HSCT) and solid organ transplant (SOT) recipients, who are highly susceptible to severe CMV‐related complications. Resistance to antiviral drugs should be suspected when CMV viral load increases by more than 1 log_10_ IU/mL or fails to decline after at least 2 weeks of antiviral therapy. CMV resistance is identified by detecting specific mutations in genes encoding antiviral targets; the development of resistance is often closely related to the antiviral agent administered in each clinical case [1, 2].

Antiviral resistance mutation (ARM) in the UL97 gene are a major mechanism of CMV resistance to ganciclovir (GCV), valganciclovir (VGCV), and ARM conferring varying levels of resistance to MBV have also been described [3]. The majority of reported ARM in clinical series involve the UL97 kinase, with substitutions commonly occurring at positions M460V/I, H520Q, C592G, A594V, L595S, and C603W [4]. Less frequent ARM, clustered between codons 590 and 607, have also been implicated in GCV resistance [5].

Additionally, ARM in the CMV DNA polymerase gene UL54 are associated with resistance to polymerase inhibitors, including GCV, VGCV, and nondependent viral kinases as foscarnet (FOS), and cidofovir (CDV). ARM in UL54 typically clustered in functional domains of the DNA polymerase and exhibited distinct resistance phenotypes. New ARM continues to be identified, some of which confer cross‐resistance to multiple antivirals, including FOS [5]. These ARM often emerge following prolonged antiviral exposure. The coexistence of ARM in both UL97 and UL54 can result in higher levels of GCV resistance [5].

Recently, two new CMV antivirals have been approved by the FDA. MBV is a UL97 kinase inhibitor, which is indicated for the treatment of posttransplant CMV infection or disease that is refractory (with or without genotypic resistance) to GCV, VGCV, CDV, or FOS. In addition to UL97, resistance to MBV has also been associated with mutations in the UL27 gene, which encodes a viral nuclear protein [5]. Letermovir (LET), a CMV DNA terminase complex inhibitor, is approved for prophylaxis of CMV infection in CMV‐seropositive adult HSCT recipients and high‐risk kidney transplant recipients. The viral terminase complex, comprising the UL56, UL89, and UL51 genes, plays a crucial role in the cleavage and packaging of viral genomes following DNA replication via rolling‐circle amplification [6]. LET resistance is primarily associated with mutations in UL56, although mutations in UL89 and UL51 have also been reported. Notably, UL51 mutations may enhance resistance conferred by UL56 mutations due to their low fitness cost [6]. In vitro studies have identified LET resistance mutations between codons 231 and 369 of UL56, indicating a low genetic barrier to resistance [1, 2, 7].

In clinical practice, laboratory testing for antiviral‐resistant CMV is essential, as many cases of persistent viremia are not related to antiviral resistance [1]. Due to the declining use of viral culture in routine diagnostics, genotypic testing has become the primary method for detecting antiviral resistance. For routine testing, Sanger sequencing of UL97 and UL54 (for GCV/VGCV/FOS resistance) and UL56 (for LET resistance) remains the most utilized [8]. However, in research and clinical trial settings, extended sequencing may include UL27 for MBV resistance and UL51, UL56, and UL89 for LET resistance.

In recent years, next‐generation sequencing (NGS) has emerged as a promising technique for the detection of CMV ARMs [9, 10, 11], with several studies evaluating its clinical utility and cost‐effectiveness compared to Sanger sequencing. NGS offers several advantages: (1) higher sensitivity, capable of detecting resistant variants even at viral loads as low as 500 IU/mL [9]; (2) detection of minor variants representing 5%–15% of the viral population, compared to ~20% detection limits for Sanger sequencing [8, 9]; and (3) potential for greater efficiency and cost‐effectiveness through multiplexing of genes and samples [8]. Nevertheless, most studies to date have focused only on UL97 and UL54, while resistance mutations in UL89 and UL51—although currently less frequent—may become increasingly relevant with the growing use of LET [7, 8, 9, 12]. Despite its advantages, the routine implementation of NGS in clinical laboratories remains limited due to technical complexity, longer turnaround times, the need for bioinformatics expertise, and challenges in data interpretation. Furthermore, only a few studies have applied NGS to CMV resistance testing in larger patient cohorts, and most have been restricted to analysis of UL97 and UL54 [13].

The primary aim of this study was to develop and apply a methodology for detecting ARMs in six target genes (UL27, UL51, UL54, UL56, UL89, and UL97) by NGS in transplant recipients. This approach seeks to expand the current knowledge of CMV resistance and highlight the relevance of NGS‐based ARM testing in clinical practice. Additionally, secondary objectives included exploring potential associations between ARM detection and clinical variables such as viral load, transplant type, treatment history, and patient outcomes.

## 2 Materials and Methods

### 2.1 Patients and Clinical Samples

In this retrospective study, 71 clinical samples from 68 CMV DNA‐positive transplant patients were analyzed by NGS. All samples had previously been submitted to the National Centre for Microbiology (CNM) for genotypic testing by Sanger sequencing, originating from hospitals across Spain. The samples were collected between January 2018 and April 2024. In some patients, multiple sequential clinical samples were available for analysis. We explicitly clarify that this study comprised (i) a single‐center cohort from Hospital Puerta de Hierro (*n* = 35) with detailed clinical data and (ii) a multicenter cohort from 33 patients across multiple Spanish hospitals, without associated clinical/demographic data.

The patients were divided into three groups. The first group included patients from Hospital Puerta de Hierro without clinical suspicion of CMV resistance (treatment responders; *n* = 13). The second group consisted of patients from Hospital Puerta de Hierro with clinical suspicion of antiviral resistance (*n* = 22). For all patients from Hospital Puerta de Hierro (*n* = 35), clinical records were available, allowing for the collection of risk factors, clinical conditions, and outcome data. The third group included patients with clinical suspicion of resistance whose samples had been submitted to the CNM for resistance genotyping from hospitals throughout Spain (*n* = 33); for this group, clinical or demographic data were not available due to data protection regulations under the CNM portfolio. All samples consisted of residual plasma or blood, which had been stored at −80°C prior to processing.

The median age of patients from Hospital Puerta de Hierro was 57 years. Among these, 16 patients had undergone solid organ transplantation (SOT)—including kidney (*n* = 7), lung (*n* = 5), heart (*n* = 2), and liver (*n* = 2) transplantation—and 19 patients had received HSCT. Definitions for resistant and refractory CMV infection were applied according to standardized criteria [1]. Individual treatment regimens, viral loads, demographic data, and clinical variables for Groups 1 and 2 are summarized in Table S6.

For positive controls, CMV DNA extracted from in vitro cultured virus previously used for diagnostic purposes was included in all experiments. Negative controls consisted of plasma and blood samples that had previously tested negative for CMV by real‐time PCR.

This study was approved by the Ethics Committee of the Instituto de Salud Carlos III (CEI PI 11_2021‐v3).

### 2.2 DNA Extraction From Clinical Samples

Extraction was performed with an automatic extractor (QIAsymphony, Qiagen) with a commercial kit (QIAsymphony Virus/Bacteria Midi Kit (96), Qiagen) as per the manufacturer's instructions.

### 2.3 Enrichment of CMV DNA Through Amplification With Specific Nested‐PCR

Nested‐PCRs were designed to amplify each whole target gene based on consensus CMV sequences from GenBank. Six pairs of external primers were used in the first round of the PCR and other six internal pairs were used in the second round. Reactions were performed in Biorad C1000 Touch Thermal Cycler using Platinum SuperFi II DNA Polymerase (Thermo Fisher Scientific, Invitrogen), according to the manufacturer′s instructions with the addition of Q5 High GC Enhancer (New England Biolabs) to all reactions, in a final volume of 50 µL. PCR products were stored at −20°C until their use in the preparation of DNA library for NGS. Primer sets used for the amplification of UL27, UL51, UL54, UL56, UL89, and UL97 genes, amplicon sizes and nested‐PCR conditions, are shown in Tables S1–S3.

### 2.4 DNA Quantification and Quality Control Throughout the Workflow

CMV viral load was quantified in all clinical samples using a previously validated real‐time PCR assay routinely used at the Reference Laboratory for Immune Preventable Diseases (National Centre for Microbiology) using WHO standards [4].

Throughout the study, DNA was quantified at multiple stages, including postextraction, postnested PCR amplification, and after library preparation. Quantification was carried out using the QuantiFluor dsDNA System with a Quantus Fluorometer (Promega), following manufacturer instructions and expressed in ng/μL. Amplicon integrity was verified via agarose gel electrophoresis (1% in TAE buffer), using the FastRuler Middle Range DNA ladder (Thermo Fisher Scientific). Electrophoresis was performed at 90 V for 45 min.

Final library quality and fragment size were assessed at the Genomics Department (National Centre for Microbiology) using the Agilent 4150 TapeStation system. DNA ScreenTape or High Sensitivity ScreenTape kits (Agilent) were selected based on library concentration to determine size distribution, quality, and DNA concentration.

### 2.5 Library Pooling and DNA Library Preparation

Following the second round of nested PCR, six amplicons corresponding to the target genes were generated per clinical sample. To prepare the DNA libraries for NGS, the resulting PCR products were pooled into a single tube per clinical sample. Each pool was adjusted to a final volume of 26 µL containing 100 ng of total DNA, following the recommendations of the NEBNext Multiplex Oligos for Illumina protocol (New England Biolabs). A total of 73 libraries were generated, corresponding to the 71 clinical samples and two additional controls (positive and negative). Library construction was then carried out using the NEBNext Multiplex Oligos for Illumina (Dual Index Primers Set 1 and 2), which allows multiplexing of up to 96 samples. Library preparation was performed according to the manufacturer's instructions. Final quantification of the DNA libraries was determined prior to sequencing.

### 2.6 Sanger Dideoxy Sequencing

Briefly, Sanger dideoxy sequencing was performed as detailed in Tarragó et al. [14] and Gómez et al. [15]. Afterwards, all sequences were downloaded, aligned, and translated by MEGA 7 software using the UL54 and UL97 gene sequences from Human Herpesvirus 5 (Merlin strain) as reference sequence (NCBI Reference Sequence: NC_006273.2).

### 2.7 NGS and Bioinformatic Analysis of Raw Data

Sequencing is carried out by the Genomics Unit of the ISCIII using NextSeq 500 Illumina platform with NextSeq 500 cartridges, which have a capacity up to 130 million reads, performing 2 reads per 150 base pair fragments. The limit of detection to avoid false positive results will be defined at 5% according to a previous study [16].

The raw FASTQ sequences obtained were sent to the Bioinformatics Unit of the ISCIII for processing and analysis by the nf‐core/viralrecon bioinformatics pipeline. NGS data were processed using the viralrecon pipeline (nf‐core/BU‐ISCIII, implemented in Nextflow DSL2 with Docker/Singularity containers) to ensure full reproducibility and traceability of computational steps.

The workflow included the following stages:

(1) Quality control of raw reads with FastQC and trimming of adapters and low‐quality bases with fastp; (2) Removal of host reads (Illumina data only) using Kraken2; (3) Read alignment against the CMV reference genome (GenBank accession NC_006273.2) using Bowtie2, followed by sorting and indexing with SAMtools; (4) For amplicon data, primer trimming with iVar; duplicate marking with Picard; and calculation of coverage metrics with mosdepth; (5) Variant calling with iVar variants (amplicon data) or BCFTools (metagenomic data), using a minimum variant frequency threshold of 5%, minimum base quality of 20, and minimum coverage of 10×. Variants below these thresholds were excluded. (6) Consensus sequence generation with iVar consensus, requiring a minimum allele frequency of 50% to call a base, otherwise reporting an “N”; (7) Functional annotation of variants with SnpEff and SnpSift to determine amino acid changes and potential impact on antiviral resistance genes; (8) Genome assembly quality assessment with QUAST, and coverage plots across the genome for visualization of sequencing depth; (9) Summary reports integrating all QC and analysis steps were produced with MultiQC. All steps were executed using containerized environments with fixed tool versions to guarantee reproducibility. This pipeline is publicly available at https://github.com/BU-ISCIII/viralrecon.

### 2.8 Characterization of ARM

Characterization of ARM versus mutation not associated to antiviral resistance was performed according to previously published data [4].

### 2.9 Statistical Analysis

All statistical analyses were performed using SPSS software version 25 (IBM Corporation Inc.). Associations among clinical conditions, risk factors, and the presence of ARM were tested using the *χ* ^2^ test or Student's *t*‐test, depending on the variable type. Fisher's exact test was used to estimate the effect of categorical variables and their interactions when sample sizes were small. Differences in viral load between groups with and without mutations were assessed using the nonparametric Mann–Whitney *U* test due to the non‐normal distribution of the data. Concordance between NGS and Sanger sequencing was evaluated by McNemar's test for paired categorical data. Unless otherwise indicated, statistical significance was established at *p* < 0.05 (95% confidence level).

## 3 Results

### 3.1 Sequencing Clinical Samples and ARM Detected by NGS

All target genes were successfully sequenced in full by NGS in 34 of the 71 clinical samples. Details for each gene are shown in Table 1.

**Table 1:** Number and rates of clinical samples successfully processed by PCR targeted NGS.

Gene targeted	UL27	UL51	UL54	UL56	UL89	UL97
*N*	55	63	62	69	55	63
%	77.5	88.7	87.3	97.2	87	88.7

**Figure 1:**
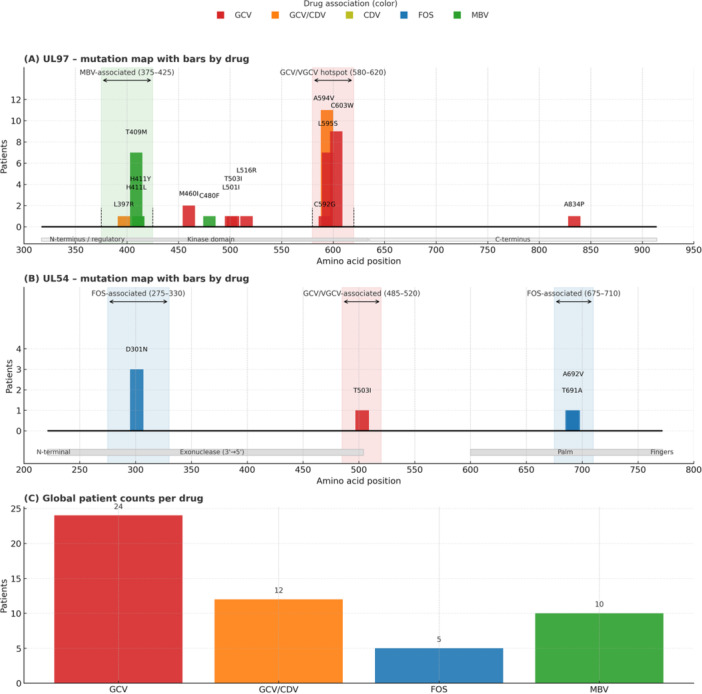
UL97 and UL54 resistance mutation maps with patient counts (targeted NGS) and global drug totals. Encoding: bars above the gene show cumulative patient counts per mutation; color encodes associated drug (GCV/VGCV ± CDV, FOS, MBV). (A) and (B) use constant‐width bars; labels are separated with manual adjustments for UL97 A594V and C603W to avoid overlap. Hotspot windows expanded: UL97 MBV (aa 375–425) and GCV/VGCV (aa 580–620); UL54 FOS (aa 275–330, 675–710) and GCV/VGCV (aa 485–520). Functional domains: UL97 N‐terminus/regulatory, kinase domain (aa 329–634), C‐terminus; UL54 exonuclease 233–504; polymerase palm 600–770 and 824–979, fingers 771–823, thumb 980–1166. Counts aggregated at patient level. (C) Global patient counts per drug.

The most frequent ARM identified by NGS were A594V (*n* = 11), C603W (*n* = 9), L595S (*n* = 7), T409M (*n* = 7), and D301N (*n* = 3). These substitutions are located in the UL97 protein kinase, a key target of GCV, and have been consistently associated with reduced susceptibility or resistance to this drug. In particular, A594V and L595S are among the most prevalent resistance mutations described worldwide and are strongly linked to clinical failure of GCV therapy in transplant recipients. C603W also confers significant GCV resistance, although it is less frequent in clinical cohorts. T409M has been reported to reduce GCV susceptibility, often appearing in combination with other UL97 mutations and contributing to cumulative resistance. D301N has been described as a resistance‐associated mutation with moderate impact, potentially contributing to reduced GCV efficacy when present alongside other substitutions. The detection of these well‐characterized ARM underscores the clinical significance of NGS in identifying both dominant and minority resistant variants that may compromise antiviral treatment in immunosuppressed patients. ARM cohort are summarized in Table 2. ARM detected by NGS and involved drugs are detailed in Figure 1A for UL97, Figure 1B for UL54, and Figure 1C for global drug totals.

**Table 2:** ARM cohort summary.

Cohort/denominator	Any ARM	UL97 ARM	UL54 ARM	UL54 + UL97 (co‐occur)	UL54 only
3.2 — Nonsuspicion (patients *n* = 13; samples *n* = 16)	3	3	0	0	0
3.3 — Suspicion (samples *n* = 55)	34	33	5	4	1

### 3.2 ARM in Transplant Recipients Without Clinical Suspicion of Resistance

We analyzed 16 samples from 13 patients without a priori suspicion of resistance (Hospital Puerta de Hierro). At the patient level, 3/13 (23.1%) carried UL97 ARM by NGS while no UL54 ARM were detected (Table 3). Importantly, in this cohort, Sanger reported S/S in all cases, including the three UL97‐ARM patients, underscoring the added yield of NGS for unsuspected resistance. Full sample‐level results are provided in Table S4. Several samples showed indeterminate calls in UL97 due to low/ambiguous coverage as shown in Table S5.

**Table 3:** Top ARM counts by gene in the nonsuspicion cohort.

Gene	ARM	Patients (*n*)	Samples (*n*)
UL54	— (none detected)	0	0
UL97	A594V	2	2
UL97	C603W	1	1

### 3.3 ARM in Transplant Recipients With Clinical Suspicion of Resistance

We sequenced 55 clinical samples (55 patients) with suspected resistance. Any genotypic resistance by NGS (UL97 and/or UL54) was found in 34/55 (61.8%) samples. UL97 ARM were detected in 33/55 (60.0%), and UL54 ARM in 5/55 (9.1%). UL54 ARM co‐occurred with UL97 ARM in all but one sample (the exception carried UL54 D301N with UL97 susceptible). The most frequent UL97 ARM were A594V (*n* = 9), C603W (*n* = 8), and L595S (*n* = 7). For UL54, D301N was observed in three samples. ARM counts by gene in the suspicion cohort are shown in Table 4. Full sample‐level results are provided in Table S5.

**Table 4:** Top ARM counts by gene in the suspicion cohort.

UL97 ARM	*n*	UL54 ARM	*n*
A594V	9	D301N	3
C603W	8	A692V	1
L595S	7	T503I	1
T409M	7	T691A	1
H411Y	2		
M460I	2		
A834P	1		
C480F	1		
C592G	1		
H411L	1		
L397R	1		
L501I	1		
L516R	1		

### 3.4 ARM Detected by NGS Versus Sanger Sequencing

All ARM detected by Sanger sequencing were also identified by NGS. In contrast, NGS detected a total of 51 ARM in 37 patients (54.4%), whereas Sanger sequencing detected only 15 ARM in 8 patients (11.8%). In 31 patients, no ARM were detected by either method. NGS identified significantly more patients with ARM than Sanger sequencing (McNemar's test: *χ* ^2^≈27.03, *p* < 0.00001).

ARM analysis of the UL54 gene revealed a total of six ARM in five patients detected by NGS, of which only three ARM in two patients were identified by Sanger sequencing. The most common UL54 mutation detected by NGS was D301N (*n* = 3), which was not detected by Sanger.

For the UL97 gene, NGS identified 45 ARM in 36 patients, while Sanger sequencing detected only 12 ARM in 6 patients. The most frequently detected mutations by NGS were A594V (*n* = 11), C603W (*n* = 9), L595S (*n* = 7), T409M (*n* = 7), followed by H411Y/L (*n* = 3), M460I (*n* = 2), with the remaining ARM each detected once. Notably, most ARM were detected exclusively by NGS, with Sanger sequencing missing several key mutations, including C603W (*n* = 9), D301N (*n* = 3), and C592G (*n* = 1).

Finally, considering detection rates across 17 distinct ARM types (Table 5), a McNemar's test was performed to compare the two methods. NGS detected all 17 ARM, while Sanger sequencing detected only 13. Four mutations were identified by NGS but missed by Sanger. McNemar's test indicated that this difference was not statistically significant at the 0.05 level (*χ* ^2^(1, *N* = 17) = 2.25, *p* = 0.134). Note that patient‐level detection (presence/absence per patient) and mutation‐level detection (across 17 ARM types) are different analyses; the former was significant, the latter was not.

**Table 5:** ARM detected in UL54 and UL97 by NGS and Sanger sequencing.

ARM	NGS	Sanger	Drug	Resistance level		Gene
A594V	11	1	GCV, CDV	High		UL97
C603W	9	0	GCV	Moderate		UL97
T409M	7	2	MBV	High		UL97
L595S	7	1	GCV	High		UL97
H411L/Y	3	1	MBV	High		UL97
M460I	2	1	GCV	Moderate		UL97
A834P	1	1	GCV	Unknown		UL97
C480F	1	1	MBV	Moderate		UL97
C592G	1	0	GCV	Moderate		UL97
L397R	1	1	GCV, CDV	Low/moderate		UL97
L501I	1	1	GCV	Low		UL97
L516R	1	1	GCV	Low		UL97
T503I	1	1	GCV	Low		UL54
T691A	1	1	FOS	Moderate		UL54
D301N	3	0	FOS	High		UL54
A692V	1	1	FOS	High		UL54

**Figure 2:**
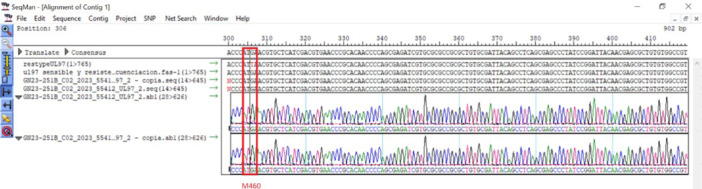
UL97 codon 460 Sanger electropherogram (Patient ID 72). A minor A peak is present under the dominant G at the third base of ATG (codon 460), consistent with M460I (ATG→ATA) but below Sanger's heterozygote calling threshold (~20%–30%), explaining the false negative by Sanger in this sample. Codon‐level translation (M460) is shown.

However, the analysis of raw Sanger sequencing data showed that minor subpopulations of ARM were not detected as for the ARM M460I, which is exemplified in the following Sample 72. Sanger chromatogram analysis ruled out minor populations considered as background or noise as shown in Figure 2.

### 3.5 Clinical Conditions of Patients and Virological Findings

**Figure 3:**
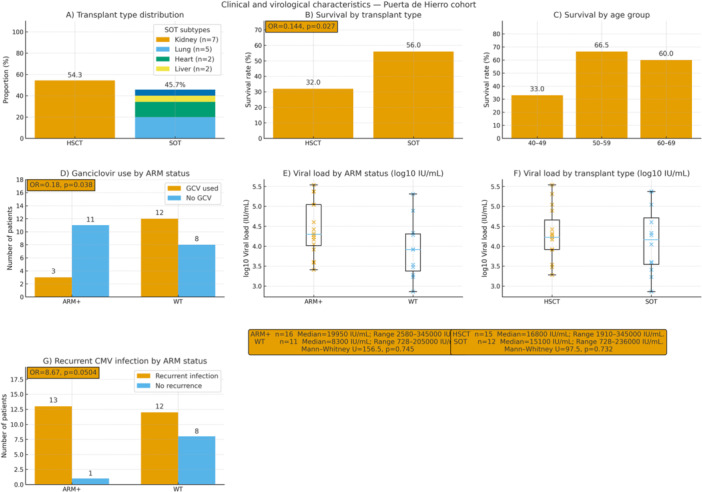
Clinical and virological characteristics of the Puerta de Hierro cohort. (A) Transplant type distribution. Of 35 patients, 19 underwent hematopoietic stem cell transplantation (HSCT) and 16 solid organ transplantation (SOT), including kidney (*n* = 7), lung (*n* = 5), heart (*n* = 2), and liver (*n* = 2). (B) Survival by transplant type. Survival was higher in SOT than HSCT recipients (56% vs. 32%; OR = 0.144, *p* = 0.027, Fisher's exact test). (C) Survival by age group. Survival rates were 33% (40–49 years), 66% (50–59 years), and 60% (60–69 years). (D) Ganciclovir exposure by ARM status. Ganciclovir use was less frequent in ARM‐positive patients (21%, 3/14) than ARM‐negative (60%, 12/20) (OR = 0.18, *p* = 0.038, Fisher's exact test). (E) Viral load by ARM status (log_10_ IU/mL). Median viral load was 83 000 IU/mL in ARM‐positive and 307 500 IU/mL in wild‐type (WT) samples; distributions did not differ (Mann–Whitney *U* = 156.5, *p* = 0.745). Medians and test statistics are shown in the panel's summary box. (F) Viral load by transplant type (log_10_ IU/mL). Median viral load was 19 000 IU/mL in HSCT and 24 000 IU/mL in SOT recipients; no significant difference (Mann–Whitney *U* = 166.0, *p* = 0.632). Medians and test statistics are shown in the panel's summary box. (G) Recurrent CMV infection by ARM status. Recurrent infection occurred more often in ARM‐positive than ARM‐negative patients (93% [13/14] vs. 60% [12/20]; OR = 8.67, *p* = 0.0504). ARM, antiviral resistance mutation; CDV, cidofovir; FOS, foscarnet; GCV, ganciclovir; HSCT, hematopoietic stem cell transplantation; LET, letermovir; MBV, maribavir; NGS, next‐generation sequencing; SOT, solid organ transplantation; VGCV, valganciclovir; WT, wild type.

Among 68 study patients, detailed clinical records were available for 35 from Hospital Universitario Puerta de Hierro (median age 57 years; 80% male). Transplant procedures included HSCT (*n* = 19) and SOT (*n* = 16)—kidney (*n* = 7), lung (*n* = 5), heart (*n* = 2), and liver (*n* = 2) (Figure 3A). Based on clinical criteria, 22/35 (63%) were suspected of antiviral resistance; ARM were detected in 16 patients (UL97, *n* = 16; UL54, *n* = 2), including two carrying mutations in both genes. Of the 13 patients without initial suspicion, 3 harbored ARM (all in UL97).

Overall survival was 43%, with substantially lower survival in HSCT than SOT recipients (32% vs. 56%; OR = 0.37, *p* = 0.027; Fisher's exact; Figure 3B). Survival by age group was 33% (40–49 years), 66% (50–59 years), and 60% (60–69 years) (Figure 3C).

Antiviral exposure patterns differed by resistance status. GCV use was significantly less frequent among ARM‐positive than ARM‐negative patients (21% [3/14] vs. 60% [12/20]; OR = 0.18, *p* = 0.038), consistent with early discontinuation/switching in nonresponders (Figure 3D). VGCV showed a nonsignificant trend toward less frequent use in ARM‐positive patients (36% vs. 65%; *p* = 0.163). No significant associations were observed for other agents (FOS, CDV, LET; all *p* > 0.5). Maribavir (MBV) was only used in ARM‐positive patients (2/14).

Viral load analyses included patients with available NGS and viral load data, excluding indeterminate results. ARM‐positive vs. WT had similar distributions (median 4.92 log_10_ IU/mL vs. 5.49 log_10_ IU/mL; Mann–Whitney *U* = 156.5, *p* = 0.745), with medians and test statistics displayed in the summary box of Figure 3E. By transplant type, HSCT versus SOT viral loads were also comparable (median 4.28 log_10_ IU/mL vs. 4.38 log_10_ IU/mL; Mann–Whitney *U* = 166.0, *p* = 0.632; see summary box in Figure 3F).

Recurrent CMV infection was more frequent in ARM‐positive than ARM‐negative patients (93% [13/14] vs. 60% [12/20]; OR = 8.67, *p* = 0.0504), suggesting a relationship between resistance development and recurrence (Figure 3G).

Full patient‐level demographics, treatments, and virological results are shown in Table S6.

## 4 Discussion

The emergence of ARM in CMV poses a significant threat to transplant recipients, particularly those undergoing HSCT or SOT, where immunosuppression increases susceptibility to viral reactivation and complicates treatment [1, 2]. Traditionally, ARM detection has relied on Sanger sequencing of the UL54 and UL97 genes. However, this approach must be reconsidered with the expanded use of MBV and LET, which target additional viral genes beyond UL54 and UL97.

Moreover, Sanger sequencing is limited in sensitivity, particularly for detecting minority resistant subpopulations, prompting the integration of NGS into clinical practice. NGS provides broader genomic coverage and enhanced detection capacity [17]. In this study, the ability of NGS to detect minority ARM populations was exemplified by the identification of the M460I mutation in one sample. This mutation was undetectable by Sanger sequencing due to its frequency falling below the detection threshold (< 30%) and being masked by the dominant wild‐type variant. This finding underscores the limitations of conventional genotyping methods and highlights the clinical utility of deep sequencing for early resistance detection and appropriate antiviral selection, particularly in complex cases where minor variants may expand under drug pressure [9].

Thanks to the capabilities of NGS, the targeted PCR approach developed in this study allowed for the sequencing of entire genes, in contrast to the fragment‐based sequencing typical of Sanger methods. This enabled the creation of a comprehensive database to support improved surveillance of ARM and polymorphisms across complete genes. Our findings demonstrated high NGS coverage across all six targeted CMV genes, ranging from 77.5% for UL27 to 97.2% for UL56. This high coverage affirms the feasibility of applying NGS routinely, consistent with previous studies in which deep sequencing enabled robust genotypic surveillance even at relatively low viral loads [5].

For UL54 and UL97 specifically, NGS enabled the sequencing of nine and six additional patients, respectively, compared to Sanger sequencing. Overall, NGS detected ARM in 29 patients that were missed by Sanger sequencing. Conversely, Sanger did not identify any additional ARM undetected by NGS. In total, NGS detected ARMs in 54.4% of patients, compared to 11.8% by Sanger sequencing. These results align with previous studies demonstrating the superior sensitivity of NGS, particularly for mutations present at frequencies below 30% [8, 9], which are often missed by Sanger's known limitations in detecting minority variants [11].

The mutational landscape observed was consistent with prior literature. In the UL97 gene, A594V, C603W, and L595S were the most frequently identified mutations, all associated with resistance to GCV and VGCV, as previously reported [4, 13]. Less common mutations such as T409M, M460I, and C592G were also detected, though at lower frequencies compared to earlier studies [9, 18]. A notable concentration of mutations was found between codons 590–607 in UL97, a well‐established hotspot for resistance that preserves viral fitness [19]. In UL54, D301N was the most commonly detected ARM, consistent with prior reports from transplant cohorts treated with DNA polymerase inhibitors [10].

A particularly significant finding was the detection of ARM in 23% of transplant recipients without clinical suspicion of resistance, identified exclusively by NGS. Notably, C603W and A594V were found in three patients who were responding to therapy at the time of sampling. These findings suggest that resistance mutations can be present even in the absence of overt treatment failure, reinforcing the need for broader implementation of resistance surveillance strategies [11].

Among patients with clinical suspicion of resistance, virological resistance was confirmed by NGS in 34 patients (61.8%), predominantly involving UL97. ARMs were significantly more prevalent in UL97 than in UL54. When UL54 mutations were present, they frequently co‐occurred with UL97 mutations, consistent with previous studies [9, 20]. Since FOS is typically used as second‐line therapy following GCV failure, the detection of FOS resistance in at least five patients significantly limited available treatment options in those cases. Additionally, ARMs associated with MBV resistance, such as T409M, M411Y, and C480F, were identified in eight patients, reflecting the growing clinical use of this newer antiviral agent.

From a clinical standpoint, survival outcomes were affected by transplant type. As reported in prior studies, HSCT recipients exhibited lower survival rates compared to SOT recipients, highlighting the vulnerability of this population to severe CMV disease [21]. However, ARM presence was not statistically associated with mortality, viral load, or patient age in our cohort, consistent with observations that ARM detection alone does not independently predict clinical outcomes without considering additional host and viral factors [18].

Interestingly, GCV use at the time of sampling was significantly less frequent among patients with ARMs (OR = 0.18, *p* = 0.038), likely reflecting early discontinuation due to clinical nonresponse and switching to alternative antivirals before resistance testing. A similar trend was observed for VGCV (OR = 0.30, *p* = 0.163), though it did not reach statistical significance [22]. MBV was exclusively used in ARM‐positive patients, consistent with its indication as a salvage therapy. Although not statistically significant (OR≈6.7, *p* = 0.162), this finding aligns with its targeted use in resistant infections.

Recurrent CMV infection was more frequent among ARM‐positive patients (93% vs. 60%), with a borderline significant association (OR = 8.67, *p* = 0.0504), supporting the hypothesis that prolonged viral replication increases the risk of resistance development [22].

Finally, NGS's principal practical advantage over Sanger is high‐throughput multiplexing: in our protocol, six CMV genes were amplified and pooled per sample—logistically impractical and cost‐prohibitive with conventional methods. In urgent single‐case scenarios, Sanger (UL97/UL54) can return results in ~1–2 working days, whereas targeted NGS typically requires ~2–4 working days including library preparation, sequencing, and reporting; with batching and streamlined bioinformatics, this gap narrows. When multiplexed across genes and samples, per‐sample reagent costs for NGS become comparable to Sanger, which grows less efficient as the number of loci or repeat tests increases. Key barriers to routine NGS adoption include the need for batching to sustain cost‐effectiveness, preanalytical constraints (adequate viral load and amplicon performance), specialized bioinformatics/reporting, and requirements for assay validation, external quality assessment, and reimbursement. Despite these hurdles, NGS offers broader gene coverage (beyond UL97/UL54, e.g., UL27/UL51/UL56/UL89) and detection of minority variants relevant for refractory disease and newer antivirals. We therefore support a hybrid workflow: prioritize targeted NGS for high‐risk contexts (e.g., HSCT, recurrent/refractory viremia, prolonged exposure to GCV/LET/MBV, rising/persistent viral load) to capture multidrug resistance and low‐frequency variants, and use Sanger for rapid single‐gene confirmation of actionable high‐frequency variants, orthogonal verification near decision thresholds, or when NGS batching is not feasible in urgent turnarounds.

## 5 Limitations

As a retrospective analysis based on residual clinical samples, certain constraints arose, particularly regarding sample volume and quality. In some cases, limited material prevented repeat sequencing or confirmation of minor variants. Although many target genes were successfully sequenced by NGS, variable genome coverage and occasional low viral loads in certain samples may have influenced detection sensitivity. Additionally, repeated cycles of freezing and thawing and the elapsed time between sample collection and analysis could affect DNA quality. An important limitation is the relatively small number of patients treated with newer antivirals such as MBV and LET, which restricted the ability to draw definitive conclusions regarding resistance to these drugs. Finally, despite the comprehensive clinical data collection from patients at Hospital Puerta de Hierro, the retrospective design inherently limits the ability to establish causality between the presence of ARM and clinical data. Moreover, patients with clinical suspicion of resistance whose samples had been submitted to the CNM for resistance genotyping from hospitals throughout Spain (excluding Hospital Puerta de Hierro), clinical or demographic data were not available due to data protection regulations under the CNM portfolio.

## Author Contributions

Conceptualization: David Tarragó. Methodology: Salvador Alemán, Juan Camacho, Vanessa Recio, and Estrella Ruiz. Validation: David Tarragó. Formal analysis: David Tarragó. Investigation: David Tarragó, Pilar Zamarrón, Jorge Anel, and Montserrat Enjuto. Data curation: Salvador Alemán. Original draft preparation: Salvador Alemán. Writing – review and editing: David Tarragó and Salvador Alemán. Funding acquisition: David Tarragó. All authors commented on the manuscript. All authors have read and agreed to the published version of the manuscript.

## Ethics Statement

This study did not involve human experimentation and all methods were carried out in accordance with relevant guidelines and EU regulations. All experimental protocols including the use of residual clinical specimens submitted for virological diagnosis. This study was approved by the Ethics Committee of the “Instituto de Salud Carlos III” (CEI PI 11_2021‐v3).

## Consent

Written informed consent from all subjects was obtained.

## Conflicts of Interest

The authors declare no conflicts of interest.

## Supporting information


**Supplementary Table S1:** Oligonucleotides designed in this study for Targeted Deep Sequencing. **Supplementary Table S2:** External and internal amplicon lengths for each gene. **Supplementary Table S3:** PCR conditions for amplification of target genes. **Supplementary Table S4:** ARM by NGS and Sanger sequencing in transplant recipients without clinical suspicion of resistance from “Hospital Puerta de Hierro”. ARM substitutions are indicated. “S” were samples with no ARM (susceptible genotype). “I” were indeterminate results due to insufficient or ambiguous sequencing data. Viral load was expressed in IU/mL. **Supplementary Table S5:** Detection of ARM by NGS and Sanger in transplant recipients with clinical suspicion of resistance. ARM substitutions are indicated. “S” were samples with no ARM (susceptible genotype). **Supplementary Table S6:** Demographic data, clinical conditions, type of transplant, therapy and virological findings including ARM and viral load among patients with/without suspicion of resistance from Hospital Puerta de Hierro.

## Data Availability

All data supporting the findings of this study are available within the article and its Supporting Information. De‐identified data and analysis scripts are available from the corresponding author upon reasonable request.
