# Supplementary material for: Unraveling Cytomegalovirus Drug Resistance in Transplant Patients by Targeting Deep Sequencing
Source: J Med Virol. 2025 Dec 29;98(1):e70768. doi: 10.1002/jmv.70768 (PMC12746535; doi:10.1002/jmv.70768)
Supplement: Supplementary file 1 — Supplementary Table S1: Oligonucleotides designed in this study for Targeted Deep Sequencing. Supplementary Table S2: External and internal amplicon lengths for each gene. Supplementary Table S3: PCR conditions for amplification of target genes. Supplementary Table S4: ARM by NGS and Sanger sequencing in transplant recipients without clinical suspicion of resistance from “Hospital Puerta de Hierro”. ARM substitutions are indicated. “S” were samples with no ARM (susceptible genotype). “I” were indeterminate results due to insufficient or ambiguous sequencing data. Viral load was expressed in IU/mL. Supplementary Table S5: Detection of ARM by NGS and Sanger in transplant recipients with clinical suspicion of resistance. ARM substitutions are indicated. “S” were samples with no ARM (susceptible genotype). Supplementary Table S6: Demographic data, clinical conditions, type of transplant, therapy and virological findings including ARM and viral load among patients with/without suspicion of resistance from Hospital Puerta de Hierro. [file JMV-98-e70768-s001.docx]

**Supplementary Table S1**. Oligonucleotides designed in this study for Targeted Deep Sequencing

| Name | Oligonucleotide (5′–3′) |
| --- | --- |
| UL27 ext F | CCCAACTGAAAAGGTTGGCG |
| UL27 ext R | ACGTCAGCGAGTACGTGTTT |
| UL27 int F | GATGACCCCACGGTGCTTAT |
| UL27 int R | TAGCCGCTGTCTGGCTTTAC |
| UL51 ext F | GACCGTGTCTGTCTTGAGCA |
| UL51 ext R | TGGTGAGTACGGTGGATTGC |
| UL51 int F | ACCCAACTTGAGGATACGCC |
| UL51 int R | TGCCATAGCAGCTCAGTTGT |
| UL54 ext F | CTTTATGCACGCCGAGAACG |
| UL54 ext R | TTCTTTGGACGGACGGACTG |
| UL54 int F | CTCCTCCTCGTCTCAGTCCA |
| UL54 int R | GGGACAGAAGCCCAACCTAC |
| UL56 ext F | GCCCAGCAGATAAGTGGTGT |
| UL56 ext R | TTTGTGCACCGACTCCATGT |
| UL56 int F | GCAGCAGACGTCGTATGAGA |
| UL56 int R | TCGTACGTGAGCGAGTCGAG |
| UL89 ext F | AGTTTTACGCGCCTGGGTTA |
| UL89 ext R | TGAAAGAGGCGGTCGACAAA |
| UL89 int F | TCGTCTCGTTTCTGCCGATG |
| UL89 int R | AAACATCCTCCGTAGTGGCG |
| UL97 ext F | ACCTTGGTGGACTCGGTTTC |
| UL97 ext R | GATGCGGTAGGCGTAAGACA |
| UL97 int F | GCCCTAGGAACAGGGAAGAC |
| UL97 int R | GTGCAGCAAAAAGGTGTTCA |

**Supplementary Table S2**. External and internal amplicon lengths for each gene

| Gene | External Amplicon (bp) | Internal Amplicon (bp) |
| --- | --- | --- |
| UL27 | 2278 | 1933 |
| UL51 | 1112 | 739 |
| UL54 | 4535 | 4100 |
| UL56 | 3078 | 2674 |
| UL89 | 1544 | 1496 |
| UL97 | 2481 | 2401 |

**Supplementary Table S3**. PCR conditions for amplification of target genes

| Phase | Temperature | UL27 | UL51 | UL54 | UL56 | UL89 | UL97 | Time |
| --- | --- | --- | --- | --- | --- | --- | --- | --- |
| Initial denaturation | 98°C | ✓ | ✓ | ✓ | ✓ | ✓ | ✓ | 0:30 |
| Denaturation (35×) | 98°C | ✓ | ✓ | ✓ | ✓ | ✓ | ✓ | 0:10 |
| Annealing (35×) | 60°C | ✓ | ✓ | ✓ | ✓ | ✓ | ✓ | 0:10 |
| Extension (35×) | Variable | 72°C/1 min | 72°C/0:30 min | 72°C/2 min | 72°C/1:30 min | 72°C/1 min | 72°C/1 min | – |
| Final extension | 72°C | ✓ | ✓ | ✓ | ✓ | ✓ | ✓ | 5:00 |

**Supplementary Table S4.** ARM by NGS and Sanger sequencing in transplant recipients without clinical suspicion of resistance from “Hospital Puerta de Hierro”. ARM substitutions are indicated. “S” were samples with no ARM (susceptible genotype). “I” were indeterminate results due to insufficient or ambiguous sequencing data. Viral load was expressed in IU/mL.

| Patient | **UL27** | **UL51** | **UL54** | **UL56** | **UL89** | **UL97** | **Viral load (IU/mL)** | **Sanger Seq**  **UL97/UL54** |
| --- | --- | --- | --- | --- | --- | --- | --- | --- |
| 1 | **I** | **S** | **S** | **S** | **I** | **S** | **1,7x10^4^** | S/S |
| 1 | **S** | **S** | **S** | **S** | **S** | **S** | **7,85x10^4^** | S/S |
| 1 | **I** | **S** | **S** | **S** | **S** | **S** | **1x10^4^** | S/S |
| 2 | **S** | **S** | **S** | **S** | **S** | **I** | **1,7x10^3^** | I/I |
| 2 | **S** | **S** | **S** | **S** | **S** | **S** | **3x10^3^** | S/S |
| 3 | **S** | **S** | **S** | **S** | **I** | **I** | **3,27x10^2^** | I/I |
| 4 | **I** | **S** | **S** | **S** | **I** | **I** | **<100-** | I/I |
| 5 | **I** | **S** | **S** | **S** | **I** | **S** | **<100-** | I/I |
| 6 | **I** | **I** | **S** | **S** | **I** | **I** | **<100-** | I/I |
| 7 | **S** | **S** | **S** | **S** | **S** | **S** | **1,92x10^4^** | S/S |
| 8 | **S** | **I** | **S** | **S** | **S** | **S** | **1,91x10^3^** | I/I |
| 9 | **S** | **S** | **S** | **S** | **S** | **I** | **2,16x10^4^** | S/I |
| 10 | **I** | **I** | **S** | **S** | **S** | **C603W** | **2,58x10^3^** | S/S |
| 11 | **S** | **I** | **S** | **S** | **S** | **A594V** | **4,04x10^3^** | S/S |
| 12 | **S** | **S** | **S** | **S** | **I** | **S** | **7,28x10^2^** | I/I |
| 13 | **S** | **S** | **S** | **S** | **S** | **A594V** | **8,30 x 10^3^** | S/S |

**Supplementary Table S5.** Detection of ARM by NGS and Sanger in transplant recipients with clinical suspicion of resistance. ARM substitutions are indicated. “S” were samples with no ARM (susceptible genotype). “I” were indeterminate or unknown genotype due to insufficient or ambiguous sequencing data. Viral load is expressed in IU/mL.

| **ID** | **UL27** | **UL51** | **UL54** | **UL56** | **UL89** | **UL97** | **Sanger sequencing UL97/UL54** | **Viral load** |
| --- | --- | --- | --- | --- | --- | --- | --- | --- |
| **14** | **S** | **S** | **S** | **S** | **S** | **S** | S/S | **3,43 x 10^3^** |
| **15** | **S** | **S** | **S** | **S** | **S** | **C603W/L595S** | S/S | **1,11 x 10^5^** |
| **16** | **S** | **S** | **S** | **S** | **S** | **L595S** | S/S | **-** |
| **17** | **S** | **S** | **S** | **S** | **S** | **S** | S/S | **3,45 x 10^5^** |
| **18** | **I** | **I** | **S** | **S** | **I** | **T409M/H411Y** | S/S | **-** |
| **19** | **S** | **S** | **S** | **S** | **S** | **C603W** | S/S | **3,87 x 10^3^** |
| **20** | **S** | **S** | **S** | **S** | **I** | **C603W** | S/S | **-** |
| **21** | **I** | **S** | **I** | **S** | **S** | **S** | I/I | **-** |
| **22** | **S** | **S** | **S** | **S** | **S** | **S** | S/S | **-** |
| **23** | **S** | **S** | **D301N** | **S** | **I** | **S** | S/S | **9,02 x 10^3^** |
| **24** | **S** | **S** | **S** | **S** | **S** | **S** | S/S | **9,37 x 10^4^** |
| **25** | **S** | **S** | **S** | **S** | **S** | **S** | S/S | **1,31 x 10^4^** |
| **26** | **S** | **S** | **S** | **S** | **I** | **S** | S/S | **1,55 x 10^4^** |
| **27** | **I** | **S** | **S** | **S** | **I** | **S** | S/S | **1,82 x 10^4^** |
| **28** | **I** | **S** | **S** | **S** | **I** | **A594V** | S/S | **-** |
| **29** | **S** | **S** | **S** | **S** | **S** | **A594V** | S/S | **1,83 x 10^4^** |
| **30** | **S** | **S** | **S** | **S** | **S** | **S** | S/S | **3,46 x 10^4^** |
| **31** | **S** | **S** | **S** | **S** | **S** | **S** | S/S | **2,69 x 10^4^** |
| **32** | **S** | **S** | **S** | **S** | **S** | **A594V** | **A594V-UL97/S** | **7,67 x 10^4^** |
| **33** | **S** | **S** | **I** | **S** | **S** | **A594V** | I/I | **8,30 x 10^3^** |
| **34** | **S** | **I** | **S** | **S** | **S** | **C480F/L595S** | **C480F-UL97/S** | **2,36 x 10^5^** |
| **35** | **S** | **S** | **S** | **S** | **S** | **T409M** | **T409M-UL97/S** | **9,34 x 10^4^** |
| **36** | **S** | **S** | **S** | **S** | **S** | **T409M/H411Y** | S/S | **-** |
| **38** | **S** | **S** | **S** | **S** | **S** | **L501I/T503I/L516R/A834P** | **L501I/T503I/L516R/A834P-UL97/S** | **1,12x10^4^** |
| **39** | **S** | **S** | **S** | **S** | **S** | **L397R/T409M/H411L/M460I** | **L397R/T409M/H411L/M460I-UL97/S** | **-** |
| **40** | **S** | **S** | **S** | **S** | **S** | **A594V** | S/S | **-** |
| **41** | **I** | **S** | **S** | **S** | **S** | **L595S** | S/S | **5,18x10^5^** |
| **42** | **S** | **S** | **S** | **S** | **I** | **I** | I/I | **-** |
| **43** | **S** | **S** | **S** | **S** | **S** | **A594V** | S/S | **8,19x10^5^** |
| **44** | **S** | **I** | **S** | **S** | **S** | **C603W** | S/S | **2,09x10^4^** |
| **45** | **S** | **S** | **S** | **S** | **S** | **S** | S/S | **2,05x10^5^** |
| **46** | **S** | **S** | **S** | **S** | **S** | **A594V** | S/S | **1,47x10^4^** |
| **47** | **S** | **S** | **T503I** | **S** | **S** | **C603W** | **S/T503I-UL54** | **4,01x10^4^** |
| **48** | **S** | **S** | **S** | **S** | **S** | **L595S** | S/S | **1,39 x 10^5^** |
| **49** | **S** | **S** | **S** | **S** | **S** | **C603W** | S/S | **1,73 x 10^4^** |
| **50** | **S** | **S** | **S** | **S** | **S** | **C603W** | S/S | **1,9 x 10^4^** |
| **51** | **S** | **S** | **D301N** | **S** | **S** | **L595S** | **L595S-UL97/S** | **-** |
| **52** | **S** | **S** | **T691A/A692V** | **S** | **S** | **A594V** | **S/T691A/A692V-UL54** | **5,52 x 10^4^** |
| **53** | **S** | **S** | **I** | **S** | **S** | **T409M** | I/I | **2,65 x 10^4^** |
| **54** | **I** | **S** | **S** | **S** | **S** | **A594V** | S/S | **1,68 x 10^4^** |
| **56** | **S** | **S** | **S** | **S** | **S** | **S** | S/S | **-** |
| **57** | **I** | **S** | **I** | **S** | **S** | **L595S** | I/I | **-** |
| **59** | **S** | **S** | **S** | **S** | **I** | **S** | S/S | **-** |
| **61** | **S** | **I** | **I** | **S** | **S** | **C592G** | I/I | **-** |
| **62** | **S** | **S** | **S** | **S** | **S** | **I** | S/S | **-** |
| **63** | **S** | **S** | **S** | **S** | **S** | **S** | S/S | **9,14x10^3^** |
| **64** | **S** | **S** | **I** | **S** | **S** | **S** | I/I | **4,17x10^3^** |
| **65** | **S** | **S** | **S** | **S** | **S** | **S** | S/S | **1,38x10^5^** |
| **66** | **S** | **S** | **D301N** | **S** | **I** | **C603W** | S/S | **3,64x10^2^** |
| **67** | **I** | **S** | **I** | **I** | **S** | **I** | I/I | **1,31x10^3^** |
| **68** | **S** | **S** | **I** | **S** | **S** | **S** | I/I | **2,10x10^4^** |
| **69** | **S** | **S** | **S** | **S** | **S** | **T409M** | S/S | **1,98x10^4^** |
| **70** | **I** | **S** | **S** | **S** | **I** | **T409M** | S/S | **1,43x10^4^** |
| **71** | **I** | **S** | **S** | **I** | **S** | **S** | S/S | **1,47x10^4^** |
| **72** | **S** | **S** | **I** | **S** | **S** | **M460I** | I/S | **1,49x10^4^** |

**Supplementary Table S6.** Demographic data, clinical conditions, type of transplant, therapy and virological findings including ARM and viral load among patients with/without suspicion of resistance from Hospital Puerta de Hierro.

| **ID** | **VIRAL LOAD UI/mL** | **TYPE OF TRANSPLANT** | **GENDER** | **AGE** | **DEATH** | **GCV** | **VAL** | **FOS** | **CDV** | **MBV** | **LET** | **PROLONGATED CMV EXPOSURE** | **CMV RECURRENT INFECTIONS** | **UL54**  **NGS** | **UL97**  **NGS** | **UL54**  **SANGER** | **UL97**  **SANGER** |
| --- | --- | --- | --- | --- | --- | --- | --- | --- | --- | --- | --- | --- | --- | --- | --- | --- | --- |
| **1** | **7,85x10^4^** | **HSTC** | **W** | **34** | **Y** | **N** | **Y** | **Y** | **N** | **N** | **N** | **Y** | **Y** | **S** | **S** | **S** | **S** |
| **2** | **1,7x 10^3^** | **SOT** | **M** | **72** | **N** | **Y** | **Y** | **N** | **N** | **N** | **N** | **Y** | **Y** | **S** | **S** | **S** | **S** |
| **3** | **3x10^3^** | **HSTC** | **M** | **69** | **Y** | **N** | **N** | **N** | **N** | **N** | **N** | **Y** | **N** | **S** | **I** | **S** | **S** |
| **4** | **3,27x10^2^** | **HSTC** | **M** | **83** | **Y** | **N** | **N** | **N** | **N** | **N** | **N** | **N** | **N** | **S** | **I** | **I** | **I** |
| **5** | **3x10^3^** | **HSTC** | **M** | **69** | **Y** | **N** | **N** | **N** | **N** | **N** | **N** | **Y** | **N** | **S** | **S** | **S** | **S** |
| **6** | **7,85x10^4^** | **HSTC** | **W** | **62** | **N** | **N** | **N** | **N** | **Y** | **N** | **N** | **Y** | **Y** | **S** | **I** | **I** | **I** |
| **7** | **1,92x10^4^** | **HSTC** | **W** | **32** | **N** | **N** | **N** | **N** | **N** | **N** | **N** | **N** | **N** | **S** | **S** | **I** | **I** |
| **8** | **1,91x10^3^** | **HSTC** | **W** | **64** | **N** | **N** | **N** | **N** | **N** | **N** | **N** | **N** | **N** | **S** | **S** | **I** | **I** |
| **9** | **2,16x10^4^** | **SOT** | **M** | **57** | **Y** | **N** | **Y** | **N** | **N** | **N** | **N** | **Y** | **Y** | **S** | **I** | **I** | **I** |
| **10** | **2,58x10^3^** | **SOT** | **M** | **69** | **N** | **Y** | **Y** | **N** | **N** | **N** | **N** | **Y** | **Y** | **S** | **C603W** | **S** | **S** |
| **11** | **4,04x10^3^** | **SOT** | **M** | **65** | **N** | **Y** | **Y** | **N** | **N** | **N** | **N** | **Y** | **Y** | **S** | **A594V** | **I** | **I** |
| **12** | **7,28x10^2^** | **SOT** | **M** | **50** | **N** | **Y** | **Y** | **N** | **N** | **N** | **N** | **Y** | **Y** | **S** | **S** | **I** | **S** |
| **13** | **8,30 x 10^3^** | **HSTC** | **W** | **62** | **N** | **N** | **N** | **N** | **Y** | **N** | **N** | **Y** | **Y** | **S** | **A594V** | **S** | **S** |
| **14** | **3,43 x 10^3^** | **HSTC** | **M** | **56** | **Y** | **Y** | **N** | **N** | **Y** | **N** | **N** | **Y** | **Y** | **S** | **S** | **S** | **S** |
| **15** | **1,11 x 10^5^** | **HSTC** | **M** | **68** | **Y** | **N** | **N** | **N** | **N** | **Y** | **N** | **N** | **N** | **S** | **C603W/L595S** | **S** | **S** |
| **16** | **8,30 x 10^3^** | **HSTC** | **M** | **56** | **N** | **Y** | **N** | **N** | **N** | **N** | **N** | **N** | **N** | **S** | **S** | **S** | **S** |
| **34** | **2,36 x 10^5^** | **SOT** | **M** | **63** | **N** | **Y** | **Y** | **N** | **N** | **Y** | **N** | **Y** | **Y** | **S** | **C480F /L595S** | **S** | **C480F** |
| **35** | **3,45 x 10^5^** | **HSTC** | **M** | **52** | **Y** | **N** | **Y** | **Y** | **Y** | **N** | **Y** | **Y** | **Y** | **S** | **T409M** | **S** | **T409M** |
| **36** | **-** | **SOT** | **M** | **31** | **N** | **Y** | **N** | **N** | **N** | **Y** | **N** | **Y** | **Y** | **S** | **T409M/H411Y** | **S** | **S** |
| **37** | **3,87 x 10^3^** | **SOT** | **M** | **55** | **N** | **Y** | **Y** | **N** | **N** | **Y** | **N** | **Y** | **Y** | **S** | **C603W** | **S** | **S** |
| **38** | **1,12x10^4^** | **SOT** | **M** | **66** | **N** | **Y** | **Y** | **N** | **N** | **N** | **N** | **Y** | **Y** | **S** | **C603W** | **S** | **S** |
| **39** |  | **HSTC** | **M** | **54** | **Y** | **Y** | **N** | **N** | **N** | **Y** | **N** | **Y** | **Y** | **S** | **L397R/T409M/H411L/M460I** | **S** | **L397R/T409M/H411L/M460I** |
| **42** | **-** | **SOT** | **M** | **57** | **Y** | **Y** | **Y** | **N** | **N** | **N** | **N** | **Y** | **N** | **S** | **I** | **I** | **I** |
| **44** | **2,09x10^4^** | **HSTC** | **W** | **56** | **Y** | **N** | **Y** | **Y** | **Y** | **Y** | **Y** | **Y** | **Y** | **S** | **C603W** | **S** | **S** |
| **45** | **2,05x10^5^** | **HSTC** | **M** | **26** | **Y** | **Y** | **N** | **Y** | **Y** | **N** | **N** | **Y** | **Y** | **S** | **S** | **S** | **S** |
| **46** | **1,47x10^4^** | **HSTC** | **M** | **37** | **Y** | **N** | **Y** | **Y** | **Y** | **N** | **N** | **Y** | **Y** | **S** | **A594V** | **S** | **S** |
| **47** | **4,01x10^4^** | **SOT** | **M** | **58** | **Y** | **Y** | **N** | **N** | **N** | **N** | **N** | **N** | **Y** | **T503I** | **C603W** | **T503I** | **S** |
| **48** | **1,11 x 10^5^** | **SOT** | **W** | **53** | **Y** | **N** | **Y** | **N** | **N** | **N** | **N** | **Y** | **N** | **S** | **L595S** | **S** | **S** |
| **50** | **1,9 x 10^4^** | **SOT** | **M** | **68** | **Y** | **N** | **Y** | **N** | **N** | **Y** | **N** | **Y** | **Y** | **S** | **C603W** | **S** | **S** |
| **51** | **-** | **SOT** | **M** | **68** | **Y** | **N** | **N** | **N** | **N** | **Y** | **N** | **Y** | **Y** | **D301N** | **L595S** | **S** | **L595S** |
| **53** | **2,65 x 10^4^** | **HSTC** | **M** | **72** | **N** | **Y** | **Y** | **N** | **N** | **Y** | **N** | **Y** | **Y** | **I** | **T409M** | **I** | **I** |
| **54** | **1,68 x 10^4^** | **HSTC** | **M** | **47** | **Y** | **Y** | **N** | **Y** | **N** | **Y** | **Y** | **Y** | **Y** | **S** | **A594V** | **S** | **S** |
| **56** | **8,30 x 10^3^** | **HSTC** | **W** | **35** | **Y** | **Y** | **N** | **Y** | **Y** | **Y** | **N** | **Y** | **Y** | **S** | **S** | **S** | **S** |
| **57** | **2,36 x 10^5^** | **SOT** | **W** | **56** | **N** | **Y** | **N** | **N** | **Y** | **Y** | **N** | **Y** | **Y** | **I** | **L595S** | **I** | **I** |
| **59** | **-** | **SOT** | **M** | **66** | **Y** | **Y** | **N** | **N** | **N** | **Y** | **N** | **Y** | **Y** | **S** | **S** | **S** | **S** |

Red ID are patients with suspicion of resistance. S (sensitive or wild type); I (indeterminate); Y (yes); N (non); M (man); W (woman).
